# Rohrer's constant, k_2_, as a factor for determining endotracheal tube obstruction

**DOI:** 10.1186/cc9576

**Published:** 2011-03-11

**Authors:** AG Flevari, N Maniatis, E Dimitriadou, M Theodorakopoulou, E Paramythiotou, N Christoforidis, A Kaziani, D Koukios, F Drakopanagiotakis, A Armaganidis

**Affiliations:** 1 Attikon University Hospital, Athens, Greece

## Introduction

The purpose of the study was to apply a method by which to measure Rohrer's constant, k_2_, in order to estimate endotracheal tube (ETT) resistance (RETT). The resistance drop across the ETT is expressed by the equation RETT = k_1 _+ k_2_V', as Rohrer described, where k_1 _is the constant of laminar flow (V') and k_2 _is the constant of turbulent flow. In our past study we graphed RETT over inspiratory V' for ETTs with inner diameters of 6.5 to 9.0 mm [1]. This graph provided us with k_1 _and k_2 _constant values, for each ETT size.

## Methods

Ten intubated patients with ETTs with difficulty in patency were included in the study. Patients were all fully sedated and mechanically ventilated, by a Siemens Servo 300 ventilator, under constant flow. Pressure data were obtained: at the proximal end of the ETT (P_proximal_), reflecting the impedance distally to the proximal end of the ETT; and at the distal end of the ETT (P_distal_), reflecting the resistance distally to the distal end of the ETT. P_distal _was recorded by an intratracheal catheter, placed 2 cm above the carinal end of the ETT. Each resistance was calculated by dividing ΔP (P_peak _- P_plateau_) by V', at every point of interest (either proximal or distal sites), using the rapid end-inspiratory occlusion method. RETT resulted from the difference: RETT = R_proximal _- R_distal_. A two-tailed *t *test (unpaired with unequal variances) was used to analyse the difference between data and the level of significance *P *was set at 0.05.

## Results

**Figure 1:**
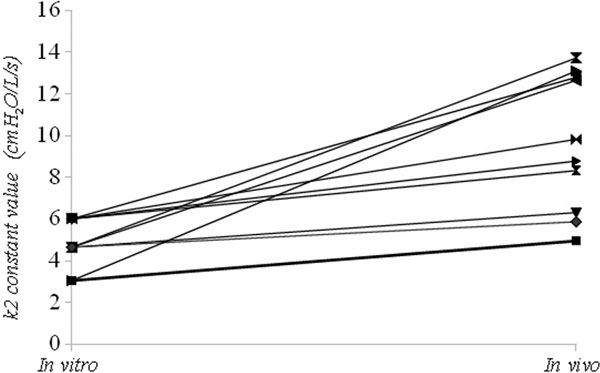
**Comparison of the k2 constant *in vivo *value with the corresponding *in vitro *k2 value**.

Ten patients (five men), with mean age of 66 ± 17 years, were tested. Figure 1 demonstrates the difference in measured k_2 _constant values compared with baseline *in vitro *values of the corresponding ETT size, for every patient. This is based on the assumption that at the moment of endotracheal intubation, the k_2 _constant has approximately the same value as the one measured *in vitro*. Figure 1 shows that the *in vivo *values were significantly higher (*P *= 0.0012).

## Conclusions

Our data suggest a significant discrepancy between predicted and *in situ *ETT resistance, raising concern for the presence of unrecognized ETT obstruction. Comparing the k_2 _constant, measured *in vivo*, with its corresponding *in vitro *value provides an estimation of ETT's resistive behaviour.
